# Instructions to Patients

**Published:** 1894-05

**Authors:** 


Instructions to Patients. By A. W. Holcombe, M. D., Kokomo, Ind.

This is a six-page pamphlet giving explicit directions to patients how to report their cases, especially if they are under treatment by correspondence.

In prescribing by letter it is very difficult to get the patient to explain his or her symptoms fully. Failure to get full indications for the proper remedy causes failure to select the curative medicine, and so failure to cure. The physician needs some printed form that he can give the patient to read, so that an intelligent report may result.

Dr. Holcombe prepared this circular for the use of his own patients, being assisted in the work by Dr. Sawyer. At the solicitation of professional friends he has decided to supply the profession.

The prices are $1.00 per hundred or 60 cents for fifty and 35 cents for twenty-five.

We cordially recommend this scheme.

